# Automated High-Definition MRI Processing Routine Robustly Detects Longitudinal Morphometry Changes in Alzheimer’s Disease Patients

**DOI:** 10.3389/fnagi.2022.832828

**Published:** 2022-06-07

**Authors:** Simon Rechberger, Yong Li, Sebastian J. Kopetzky, Markus Butz-Ostendorf, for the Alzheimer’s Disease Neuroimaging Initiative

**Affiliations:** ^1^Viscovery Software GmbH, Vienna, Austria; ^2^Biomax Informatics, Munich, Germany; ^3^School of Life Sciences, Technical University of Munich, Freising, Germany; ^4^Parallel Programming, Department of Computer Science, Technical University of Darmstadt, Darmstadt, Germany

**Keywords:** longitudinal surface based morphometry, longitudinal voxel based morphometry, SBM, VBM, HCP MMP 1.0, dementia, clinical trials, neurodegeneration

## Abstract

Longitudinal MRI studies are of increasing importance to document the time course of neurodegenerative diseases as well as neuroprotective effects of a drug candidate in clinical trials. However, manual longitudinal image assessments are time consuming and conventional assessment routines often deliver unsatisfying study outcomes. Here, we propose a profound analysis pipeline that consists of the following coordinated steps: (1) an automated and highly precise image processing stream including voxel and surface based morphometry using latest highly detailed brain atlases such as the HCP MMP 1.0 atlas with 360 cortical ROIs; (2) a profound statistical assessment using a multiplicative model of annual percent change (APC); and (3) a multiple testing correction adopted from genome-wide association studies that is optimally suited for longitudinal neuroimaging studies. We tested this analysis pipeline with 25 Alzheimer’s disease patients against 25 age-matched cognitively normal subjects with a baseline and a 1-year follow-up conventional MRI scan from the ADNI-3 study. Even in this small cohort, we were able to report 22 significant measurements after multiple testing correction from SBM (including cortical volume, area and thickness) complementing only three statistically significant volume changes (left/right hippocampus and left amygdala) found by VBM. A 1-year decrease in brain morphometry coincided with an increasing clinical disability and cognitive decline in patients measured by MMSE, CDR GLOBAL, FAQ TOTAL and NPI TOTAL scores. This work shows that highly precise image assessments, APC computation and an adequate multiple testing correction can produce a significant study outcome even for small study sizes. With this, automated MRI processing is now available and reliable for routine use and clinical trials.

## Introduction

Cross-sectional studies have shown that cognitive decline at different stages of the Alzheimer’s disease (AD) correlate with gray matter loss (Van de Mortel et al., 2021; Wu et al., 2021). At late stages of AD, a widespread reduction in brain volume was consistently found in the hippocampus, temporal pole, medial- and inferior temporal cortex, precuneus, parietal lobe and cerebellum (Chapleau et al., 2016; Dicks et al., 2019; Kang et al., 2019; Kunst et al., 2019). A particular interest, has been in the basal forebrain showing baseline differences in volume and its fiber projections between healthy aged adults and AD subjects (Teipel et al., 2011). Changes in brain volume and fiber projection were predictive for AD progression. Still, little is known about the dynamics of the neurodegenerative process and which brain areas are affected strongest by AD and differ most from typical aging (Schmitz and Nathan Spreng, 2016; Schmitz et al., 2018) or other forms of dementia (Landin-Romero et al., 2017).

Longitudinal MRI studies are a reliable way to detect brain changes at different neurodegenerative disease stages (Hua et al., 2008; Leow et al., 2009; Morra et al., 2009; Cho et al., 2013). Still, longitudinal MRI studies are relatively rare. Large between-study heterogeneity of designs and methods, differences in sample characteristics and the generally larger inter-individual variability in samples of older adults make it difficult to extract general trends. To overcome those limitations, longitudinal MRI studies require enhanced neuroimaging pipelines (Iannopollo and Garcia, 2021) and adequate statistical approaches. A very recent two-study comparison design longitudinal MRI study was able to show that it is possible to extract generalizable effects of brain atrophy in healthy aging people (Jockwitz et al., 2021).

Another source of inconsistency in longitudinal studies are varying observation intervals between baseline and follow-up assessments, which, if left uncorrected, may increase inter-individual variability. Annual percent change (APC) is a mathematical approach widely used to describe growth (Malthus, 1798; Schaechter et al., 1962) and degeneration processes (Aylward et al., 2018) but also brain morphometry changes in longitudinal studies (Dubois et al., 2015; Cavedo et al., 2017). Using a multiplicative approach for APC (Jockwitz et al., 2021) assuming a uniform brain morphometry change over a limited period of time, individual differences in observation intervals can be safely corrected.

The aim of the present study was to develop a highly precise and fully automated longitudinal morphometry pipeline that is able to detect statistically significant changes between AD patients and cognitively normal (CN) aged individuals and to safely distinguish AD from CN groups by advanced neuroimaging features. For this, we used longitudinal datasets with high image quality data from ADNI3 study (Weiner et al., 2017). We applied longitudinal ROI and voxel based morphometry (VBM) of subcortical regions and cortical subfields, such as hippocampus, amygdala and brain-stem, and complemented it by longitudinal surface based morphometry (SBM) of the cortex. For SBM, we used the latest and highest resolving surface-based brain atlas, the HCP MMP 1.0 atlas (Glasser et al., 2016) with 360 cortical ROIs.

Both methods together lead to a highly precise parcellation of the entire brain. However, the high number of group comparisons for all ROIs required for multiple testing correction of the statistical significance. Standard approaches as Bonferroni (Dunn, 1961) or even Benjamini–Hochberg (Benjamini and Hochberg, 1995) are often too pessimistic resulting in an erasure of significant effects or require unrealistically huge sample sizes. Here, we adopted a multiple testing correction approach from genome studies (Storey and Tibshirani, 2003) and showed its particular suitability for longitudinal MRI studies with high number of ROIs.

Applying the above-described imaging workflow together with the appropriate statistical group analysis revealed only three significantly differently changing ROIs by VBM analysis in AD vs. CN subjects but 22 ROI features measured by SBM. We further compared morphometric alterations in AD patients and CN subjects with cognitive testing results and found a correlation between regional atrophy and cognitive decline.

Using most precise measuring of changes in 360 cortical ROIs in high image quality data, a safe correction of individual longitudinal observation intervals and an appropriate multiple testing correction enabled us to show significant differences in brain morphometry changes correlating with cognitive decline and to clearly distinguish between AD patients and CN controls even with relatively small sample sizes.

## Materials and Methods

### Subjects

We included 25 AD patients and 25 cognitively normal (CN) aged subjects from the ADNI3 study (Weiner et al., 2017) for whom two high quality MRI scans, a baseline scan and a 1-year follow-up, were available. AD subject ages were of the range 56.6–91.2 years and CN subjects ages of 65.4–87.1 years. CN subjects were (a) free of memory complaints, (b) showed normal memory function in the Logical Memory II subscale from the Wechsler Memory Scale, (c) had a Mini-Mental State Examination (MMSE) score at or above 24, and (d) had a clinical dementia rating (CDR) of 0. The classification of AD was based on: (a) memory complaints, (b) abnormal memory function in the Wechsler test, (c) a MMSE score at or below 24, and (d) a CDR of 0.5 or greater (see ADNI General Procedure Manual, 2008, p.27^1^). T1-weighted MR images were used to perform morphometry analyses (Table 1 and Supplementary Table 1).

**TABLE 1:** Demographic table.

	Screening		Year 1	
	AD	CN	AD	CN
Age	74.6 ± 7.8	71.0 ± 4.6	75.6 ± 7.8	72.0 ± 4.6
Sex (Male/Female)	16/9	11/14	16/9	11/14
MMSE	22.76 ± 2.74	28.70 ± 3.10	21.13 ± 4.61	28.04 ± 4.56
CDR GLOBAL	0.82 ± 0.24	0.14 ± 0.42	1.14 ± 0.52	0.12 ± 0.42
FAQ TOTAL	15.00 ± 6.18	1.33 ± 5.09	17.43 ± 8.12	1.83 ± 5.94
NPI TOTAL	10.20 ± 10.15	1.12 ± 2.03	13.33 ± 13.09	2.63 ± 4.75

*All values (apart from sex) for screening visit and year 1 visit for Alzheimer’s disease patients (AD) and cognitively normal subjects (CN) are given as group averages and standard deviation. For sex, the number of males and females is given, respectively. Individual values and APOE genotypes where available are listed in Supplementary Table 1.*

### Image Quality Assurance

Systematic and manual quality assurance procedure was applied in two stages: At stage one, a after the raw image conversion from DICOM to Nifti format, all structural images were visually inspected comparing with image quality measures calculated by CAT12 toolbox (Dahnke et al., 2013). At stage two, after segmentation by CAT12, all modulated normalized gray matter longitudinal segments were verified for inter-subject homogeneity. Subjects’ homogeneity test score falling out two standard deviations of the group mean were considered as outliers. In this study, there were two subjects, one from each group, that had to be excluded from the final statistical analyses for this reason.

### Image Processing

**FIGURE 1:**
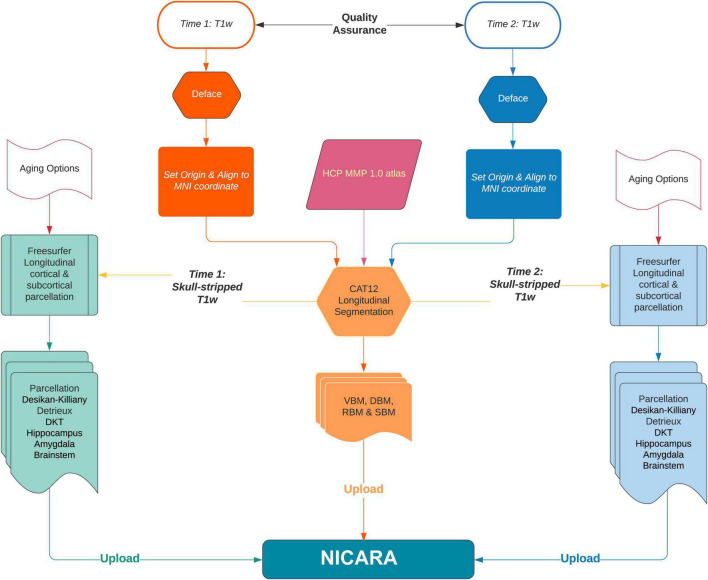
The flowchart of NICARA morphometry longitudinal pipeline processes making use of FreeSurfer, CAT12 toolbox and NICARA.

Longitudinal imaging data sets were automatically (pre-)processed with the morphometry pipeline (Figure 1) executed by NICARA*™* NeuroImaging-based Connectome Assessment in Research and Application (NICARA vers. 1.1, Biomax Informatics AG, Planegg, Germany^2^) and all results were managed and assessed by the help of NICARA. Morphometry pipelines were developed based on open-source imaging processing and analysis software and tools, such as the Computational Anatomy Toolbox (CAT12.7-r1742^3^) ran under Statistical Parametric Mapping, Version 12 (SPM12^4^), and Freesurfer 7.0^5^.

Cortical gray matter thinning as the gold-standard to detect aging and neurodegenerative diseases progression was computed longitudinally by NICARA morphometry longitudinal pipeline executing both CAT12 and Freesurfer7. Voxel based morphometry (VBM) and surface based morphometry (SBM) analysis methods were adopted to perform atlas ROI-based segmentation and parcellation. VBM could identify longitudinal volume changes at global and local brain region level. At the global level, the whole brain volume of gray matter (GM), white matter (WM) and cortical-spinal fluid (CSF) was segmented by CAT12 and estimated the total inter-cranial volume (TIV) was estimated. After the whole brain segmentation, modulated GM images were normalized to the MNI standard space with DARTEL algorithm (Ashburner, 2007) and were resampled to 1.5 × 1.5 × 1.5 mm^3^ by default CAT12 setting.

At the local brain region level, SBM used 360 ROIs defined by HCP MMP 1.0 atlas (Glasser et al., 2016) in subject’s native space by inversing the parameters of modulated GM normalization by CAT12. VBM of subcortical and specific brain region subfields was processed by Freesurfer7 recon-all longitudinal stream (Reuter et al., 2012; Iglesias et al., 2016). This approach combining with CAT12 VBM resulted in 19 subcortical ROIs by the Harvard-Oxford atlas (Frazier et al., 2005; Desikan et al., 2006; Makris et al., 2006; Goldstein et al., 2007) and 64 ROIs by hippocampal subfields (Iglesias et al., 2015b) and nuclei of the amygdala (Saygin et al., 2017), 5 ROIs by brain stem (Iglesias et al., 2015a) in one process. Brainstem subregions were computed based on longitudinal parcellated images by Freesurfer7 since there is no longitudinal pipeline for this model yet.

### Correction for Variations in Individual Longitudinal Observation Intervals

We corrected for deviations from the expected longitudinal observation interval of 1 year by using annual percent change (APC) as a measure for longitudinal changes in voxel and surface based measures. The APC was calculated from the actually observed measurements x_1_ and x_2_ and timepoints t_1_ and t_2_, respectively, by the following equation:

$$A\,P\,C=\left({\left(\frac{x_{2}}{x_{1}}\right)}^{\frac{n}{t_{2}-t_{1}}}-1\right)\cdot 100\%$$

This correction is necessary as not all study subjects returned for a second image acquisition exactly after *n* = 365 days. The annual percent change is described by a multiplicative growth and decay process that leads to the following statistical model.

### Statistical Model for Longitudinal Change in Brain Morphometry

Longitudinal imaging studies generate pairs of (or sets of more) strictly positive measurements *x*_1_, *x*_2_, of the same quantity at different time points *t*_1_, *t*_2_, such as, e.g., volumes, areas, thickness, etc. To obtain a comparable measure of change of such a variable across different spatiotemporal scales, we have to normalize with respect to starting value *x*_1_ and time difference *t*_2_ − *t*_1_.

The normalization with respect to *x*_1_ is straight forward and leads to the definition of a relative change:


(1) $$R\,C=\frac{x_{2}-x_{1}}{x_{1}}$$

This definition implicitly states that two observed relative change values are always equally severe irrespective of the baseline measurement *x*_1_, which is a reasonable assumption for neurodegeneration. We further assume that the relative differences per time unit are constant. This simplification is often used to model biological degeneration and growth processes (Schaechter et al., 1962; Bar-On et al., 2002; Aylward et al., 2018) and leads to the following exponential growth/decay model


(2) $$x\,\left(t\right)=x_{0}\,{\left(1+q\right)}^{t}$$

with *x*(*t*) being the evolving quantities over time, and *q* the growth rate for one unit of *t*.

For our purpose we are interested in expected annual changes given our measurements *x*_1_ and *x*_2_, so by evaluating equation (2) for *x* (*t*_1_) = *x*_1_, *x* (*t*_2_) = *x*_2_ and calculating the ratio of *x* (*t*_2_) and *x* (*t*_1_) we obtain


(3) $$\frac{x_{2}}{x_{1}}=\frac{x\,\left(t_{2}\right)}{x\,\left(t_{1}\right)}=\frac{x_{0}\,{\left(1+q\right)}^{t_{2}}}{x_{0}\,{\left(1+q\right)}^{t_{1}}}={\left(1+q\right)}^{t_{2}-t_{1}}$$

Or equivalently


(4) $$q={\left(\frac{x_{2}}{x_{1}}\right)}^{\frac{1}{t_{2}-t_{1}}}-1$$

In order to arrive at the *annual percentage change (APC)* formula, we have to replace the unit of time by the actual longitudinal time interval of the study *n*—in case of annual percent change by 1 year.


(5) $$A\,P\,C=\left({\left(\frac{x_{2}}{x_{1}}\right)}^{\frac{n}{t_{2}-t_{1}}}-1\right)\cdot 100\%$$

As *t*_1_ and *t*_2_ are measured in days, we need to set *n* to 365 days for APC.

Note, that some previous longitudinal studies used a linear model to evolve, e.g., brain volume changes over time by *x* (*t*) = *x*_1_ + *P* ⋅ *x*_1_ ⋅ *t*, with $P=\frac{x_{2}-x_{1}}{x_{1}\,\left(t_{2}-t_{1}\right)}$. However, simply dividing by the time difference to normalize varying observation intervals implies that the (scaled) absolute difference of *x*_1_ and *x*_2_ will be the same for all time intervals of the same length *t*_2_ − *t*_1_, which, of course, is not true and can lead, for example, to negative values of *x*(*t*) if *x*_2_ < *x*_1_. In this case, we would have *P* < 0 and by choosing larger *t* values, *x*(*t*) shrinks indefinitely, which is impossible for strictly positive quantities such as brain volume!

### Multiple Testing Correction

When testing many variables for statistically significant differences between two (or more) groups, it becomes very likely to find variables with low *p*-values, even when there is no real difference between the groups, but just random fluctuations in the variables. To account for this effect there are several techniques to correct for multiple tests. Approaches controlling the family wise error rate (FWER)—such as the Bonferroni correction (Dunn, 1961)—are very conservative. They allow practically no type I errors (false positives), while type II errors (false negatives) are extremely common. Naturally, this class of approaches is not suitable for applications with hundreds or thousands of tests, especially if the number of subjects in each group is limited.

The multiple testing correction of Benjamini and Hochberg (1995) introduced a new concept: Instead of the FWER they control and thereby introduce an upper bound of the false discovery rate (FDR), which is the rate of false positives in the set of all variables, which have been declared as significant. For analyses with a large number of tests, this is a much better approach than FWER control procedures, but still might be too conservative especially for neuroimaging studies with many ROI assessments that are tested for significance.

The approach of Storey and Tibshirani (2003) can under certain conditions increase the statistical power of a multiple testing correction by using the information that *p*-values of true null hypotheses are uniformly distributed on the unit interval, while *p*-values of false null hypotheses are concentrated near 0. Conditions for the Storey and Tibshirani approach to actually increase statistical power, are

1) A large number of individual tests,
2) *p*-values are approximately uniformly distributed near 1,
3) *p*-value distribution has a maximum near 0.

If these conditions are fulfilled, the *p*-value distribution can be used to estimate the rate π_0_ of true null hypotheses over all tests and in turn the FDR. This estimate is closer to the actual FDR than the upper bound obtained by the Benjamini–Hochberg procedure. For this study, an FDR of α = 0.05 resulted in more ROI assessments remaining significant after Storey-Tibshirani multiple testing correction compared to the Benjamini-Hochberg approach. If the above conditions are not (all) fulfilled, both multiple testing corrections are comparably pessimistic.

## Results

### Longitudinal Voxel Based Morphometry Detected Increased Hippocampus and Amygdala Decay

**FIGURE 2:**
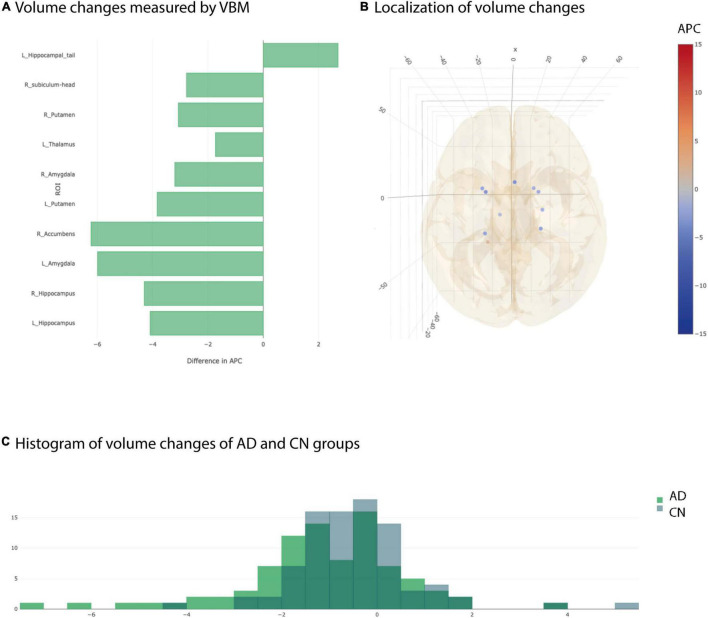
Significant differences at individual testing level of annual percent changes measured by longitudinal Voxel Based Morphometry. **(A)** Horizontal bar plot of significant differences in group averaged volume changes (longitudinal VBM) between AD and CN groups. Bars to the left indicate a stronger decrease in APC values in AD group than in CN group. **(B)** 3D lattice graph of ROIs with significant differences in APC values corresponding to panel **(A)**. Each circle is located at the center of mass of the corresponding ROI in MNI152 coordinate space. Color code indicates extend and sign of APC group differences whereas the diameter indicates absolute effect size. Brain hull was derived from a T1-weighted MR image in MNI152 standard space. **(C)** Overlaid histograms of the AD and CN group showing the group averaged APC values of all ROIs (including those with significant and non-significant differences).

We used longitudinal VBM to detect changes in brain volume over a period of 1 year of 19 subcortical ROIs according to the Harvard-Oxford atlas and of 68 ROIs of subfields of the hippocampus/amygdala and brainstem according to corresponding FreeSurfer subfield atlases. All changes are given in APC averaged over all subjects of the respective group. The longitudinal VBM statistical assessment between AD group and CN group revealed ten significant ROIs at individual testing level (Figures 2A,B). Three ROIs remained significant after multiple testing correction with Benjamini–Hochberg approach namely left and right hippocampus and left amygdala (Table 2). For the longitudinal VBM group comparison the Storey-Tibshirani approach was equally pessimistic as the Benjamini–Hochberg multiple testing correction because the preconditions of the Storey-Tibshirani method for the distribution of individual *p*-values were not fulfilled (Supplementary Figure 1).

**TABLE 2:** Significant results from VBM after multiple testing correction.

	ROI	Anatomical Classification*	Mean AD	STDV AD	Mean CN	STDV CN	p	Multiple Testing Correction	
								p_adjBH	q
Volume	L_Hippocampus	Medial temporal lobe	−4.40	3.60	–0.32	2.19	0.0000	0.0004	0.0012
	R_Hippocampus	Medial temporal lobe	−3.87	3.94	0.43	3.94	0.0003	0.0054	0.0146
	L_Amygdala	Medial temporal lobe	−7.36	6.13	–1.37	6.61	0.0017	0.0182	0.0494

For VBM volume, significant results from simple univariate t-test statistics (column p-value) remained statistically significant for left and right hippocampus and left amygdala after multiple testing correction with either Storey-Tibshirani (column q-value) or Benjamini–Hochberg (column p-adjBH-value). In this case, Storey-Tibshirani approach for multiple testing correction did not result in more significant ROIs than Benjamini–Hochberg approach. See main text for description. *Baker et al. (2018).

Considering the distribution of the average APC values of all ROIs for AD and CN group, not just the significant ones, showed two different, approximately Gaussian distributions with the AD group distribution slightly shifted to the left indicating overall smaller APC values for AD group than for CN group (Figure 2C). However, the overlap between AD and CN distributions are pretty high. That is why we complemented longitudinal VBM by longitudinal SBM.

### Longitudinal Surface Based Morphometry Showed Detailed Differences Between Alzheimer’s Disease and Cognitively Normal

We performed longitudinal SBM to detect changes over 1 year of 360 brain regions according to the HCP MMP 1.0 atlas (Supplementary Table 2; Glasser et al., 2016). All ROI-based results were given in APC. Statistical group comparisons between AD patients and CN controls were done for cortical volume, area, thickness, gyrification and depth as computed by SBM.

### Temporal Cortex Showed Accelerated Decay in Volume, Area and Thickness in Alzheimer’s Disease

**FIGURE 3:**
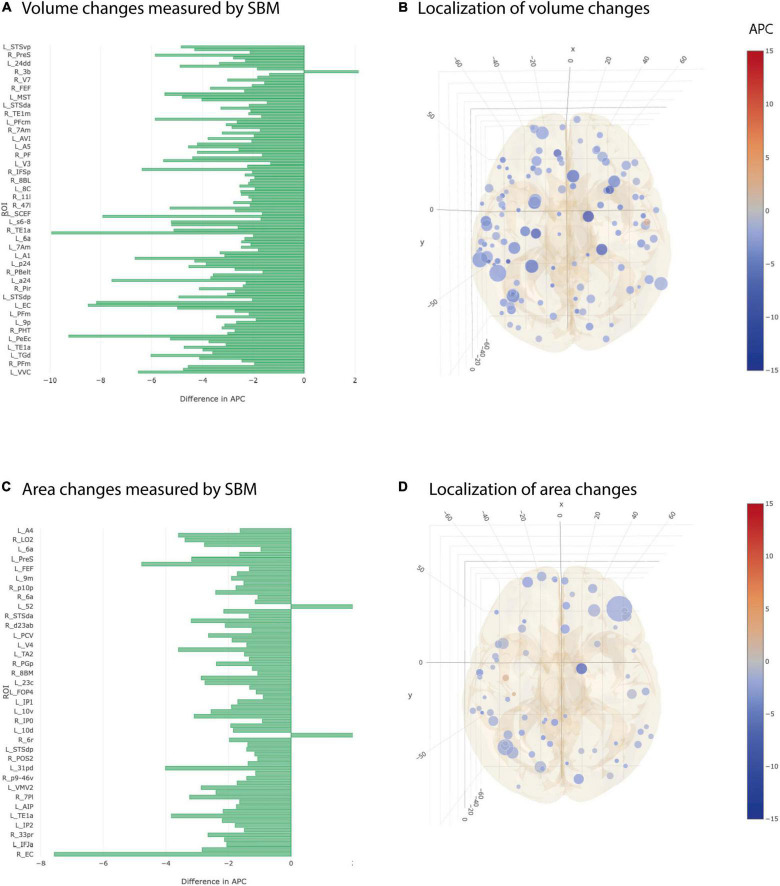
Significant differences at individual testing level of annual percent changes in cortical volume and area (longitudinal SBM). **(A)** Horizontal bar plot of differences in group averaged cortical volume changes measured by SBM between AD and CN groups. Bars to the left indicate a relatively stronger decrease in APC values in AD group than in CN group. **(B)** 3D lattice graph of ROIs with significant differences in cortical volume changes shown in APC differences corresponding to panel **(A)**. Each circle is located at the center of mass of the corresponding ROI in MNI152 coordinate space. Color code indicates extend and sign of APC group differences whereas the diameter indicates absolute effect size. Brain hull was derived from a T1-weighted MR image in MNI152 standard space. **(C)** Horizontal bar plot of differences in group averaged cortical area changes measured by longitudinal SBM between AD and CN groups. Bars to the left indicate a relatively stronger decrease in APC values in AD group than in CN group. **(D)** 3D lattice graph of ROIs with significant differences in area changes shown in APC differences corresponding to panel **(C)**.

At individual testing level, 117 ROI-based longitudinal SBM cortical volume group comparisons were significant (Figures 3A,B). After multiple testing correction, still 15 ROIs showed a significantly stronger decay in AD group compared to controls (Table 3). Significant ROIs were located in the temporal lobe, at the lateral and basal surface of the occipital lobe, temporo-parieto-occipital junction.

**TABLE 3:** Significant results from SBM after multiple testing correction.

	ROI	Anatomical Classification*	Mean AD	STDV AD	Mean CN	STDV CN	p	Multiple Testing Correction	
								p_adjBH	q
Volume	L_VVC	Basal surface of occipital lobe	−4.50	3.68	2.02	4.88	0.0000	0.0045	0.0028
	L_PH	Lateral surface of occipital lobe	−2.98	3.37	1.76	3.48	0.0000	0.0104	0.0065
	R_TGd	Temporal gyrus areas	−5.38	3.95	−0.83	3.46	0.0001	0.0264	0.0165
	R_PFm	Inferior parietal lobule and TPOJ areas	−1.99	1.86	−0.05	1.32	0.0001	0.0264	0.0165
	L_TGd	Temporal gyrus areas	−5.90	5.35	0.12	4.68	0.0001	0.0264	0.0165
	L_PF	Inferior parietal lobule and TPOJ areas	−2.93	2.30	−0.49	1.80	0.0001	0.0275	0.0172
	L_TF	Temporal areas	−3.82	3.83	0.30	3.27	0.0002	0.0325	0.0203
	L_TE1p	Temporal areas	−3.73	3.89	−0.13	2.16	0.0002	0.0330	0.0206
	L_A4	Temporal hypotenuse regions of insula and opercular cortex	−3.90	3.05	−0.83	2.53	0.0003	0.0460	0.0287
	L_FST	Lateral surface of occipital lobe	−2.44	3.00	1.55	4.21	0.0003	0.0460	0.0287
	L_TE1a	Temporal areas	−4.91	4.31	−0.20	4.35	0.0004	0.0460	0.0287
	L_PeEc	Medial temporal areas	−6.50	4.99	−1.25	4.80	0.0004	0.0498	0.0311
	R_TE2a	Temporal areas	−2.78	2.49	0.97	4.35	0.0005	0.0520	0.0325
	R_EC	Medial temporal areas	−8.84	9.59	0.42	8.60	0.0008	0.0642	0.0401
	L_FFC	Basal surface of occipital lobe	−1.89	2.99	1.11	2.93	0.0008	0.0642	0.0401
Area	R_EC	Medial temporal areas	−5.44	5.93	2.14	6.70	0.0001	0.0264	0.0165
	L_9a	Lateral superior frontal gyrus regions	−2.10	2.59	0.75	2.40	0.0002	0.0330	0.0206
	L_PH	Lateral surface of occipital lobe	−2.10	2.61	0.02	1.22	0.0006	0.0573	0.0357
	R_33pr	Anterior cingulate gyrus	−2.72	2.62	−0.06	2.57	0.0007	0.0642	0.0401
	L_IFJa	Inferior frontal gyrus regions	−1.77	1.57	0.28	2.39	0.0008	0.0642	0.0401
Thickness	R_s6-8	Superior frontal sulcus and middle frontal gyrus regions	−2.86	2.75	0.05	2.04	0.0001	0.0264	0.0165
	L_TE2p	Temporal areas	−2.06	2.68	1.11	3.27	0.0005	0.0520	0.0325

For SBM cortical volume, area and thickness, significant results from simple univariate t-test statistics (column p-value) remained statistically significant after multiple testing correction with either Storey-Tibshirani (column q-value) or Benjamini–Hochberg (column p-adjBH-value). See main text for description. *Baker et al. (2018).

Group averages of cortical area were statistically different at individual testing level in 67 ROIs and showed a stronger decay in AD group compared to CN group (Figures 3C,D). Five out of 67 ROIs remained significant after multiple testing correction. Those were located in the temporal cortex, superior and frontal gyrus, anterior cingulate cortex and lateral surface of occipital cortex (Table 3).

**FIGURE 4:**
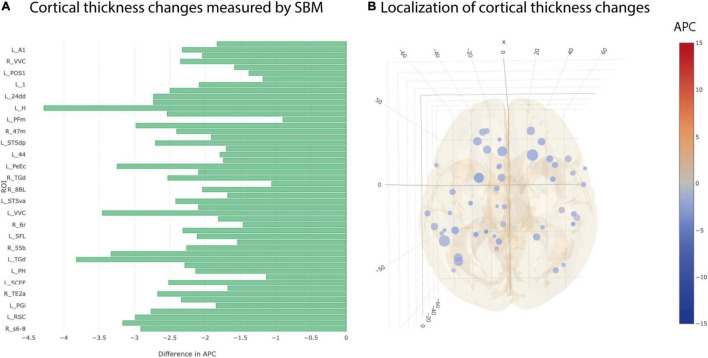
Significant differences at individual testing level of annual percent changes in cortical thickness. **(A)** Horizontal bar plot of differences in group averaged cortical thickness changes (longitudinal SBM) between AD and CN groups. Bars to the left indicate a relatively stronger decrease in APC values in AD group than in CN group. **(B)** 3D lattice graph of ROIs with significant differences in volume changes shown in APC differences corresponding to panel **(A)**. Each circle is located at the center of mass of the corresponding ROI in MNI152 coordinate space. Color code indicates group extend and sign of APC differences whereas the diameter indicates absolute effect size. Brain hull was derived from a T1-weighted MR image in MNI152 standard space.

Cortical thickness returned 50 ROIs in simple univariate statistics changing significantly between AD and CN group (Figure 4). After multiple testing correction, two ROIs in the temporal and frontal cortex remained significant (Table 3).

### Inconsistent Findings for Cortical Gyrification and Depth Changes

**FIGURE 5:**
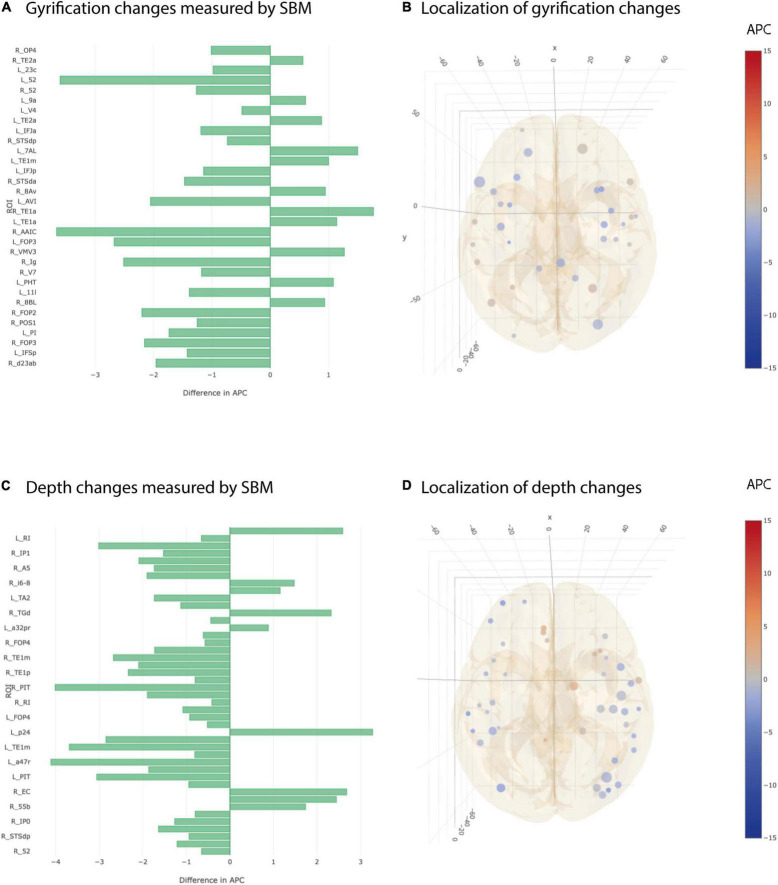
Significant differences at individual testing level of annual percent changes in cortical gyrification and depth. **(A)** Horizontal bar plot of differences in group averaged cortical gyrification changes (longitudinal SBM) between AD and CN groups. Bars to the left indicate a relatively stronger decrease in APC values in AD group than in CN group. **(B)** 3D lattice graph of ROIs with significant differences in cortical gyrification changes corresponding to panel **(A)**. Each circle is located at the center of mass of the corresponding ROI in MNI152 coordinate space. Color code indicates group extend and sign of APC differences whereas the diameter indicates absolute effect size. Brain hull was derived from a T1-weighted MR image in MNI152 standard space. **(C)** Horizontal bar plot of differences in group averaged cortical depth changes measured by longitudinal SBM between AD and CN groups. Bars to the left indicate a relatively stronger decrease in APC values in AD group than in CN group. **(D)** 3D lattice graph of ROIs with significant differences in cortical depth changes (difference in APC) corresponding to panel **(C)**.

The most heterogeneous picture of longitudinal differences between AD and CN group was seen in cortical gyrification and cortical depth. There were 21 ROIs showing a statistically significant stronger decrease in gyrification in AD vs CN group and at the same time 11 ROIs showing the opposite effect in simple univariate testing (Figures 5A,B). Additionally, we found 35 ROIs that decreased significantly stronger in cortical depth for AD group than for CN group but also 9 ROIs showing the opposite effect (Figures 5C,D). None of the observed statistical significances for cortical gyrification and depth remained significant after multiple testing correction.

### Clear Group Differences of Annual Percent Change Distribution of Cortical Volume, Area and Thickness

**FIGURE 6:**
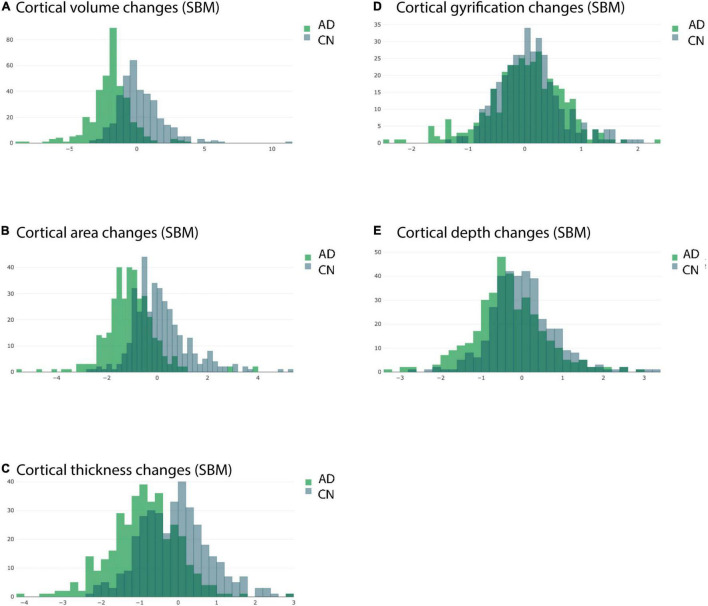
Overlaid histograms of the AD and CN group averaged APC values. Each histogram shows the group averaged APC values from longitudinal SBM of all ROIs (including those with significant and non-significant differences) from the respective group. **(A)** Changes in cortical volume measured by longitudinal SBM. **(B)** Changes in cortical area measured by longitudinal SBM. **(C)** Changes in cortical thickness measured by longitudinal SBM. **(D)** Changes in cortical gyrification measured by longitudinal SBM. **(E)** Changes in cortical depth measured by longitudinal SBM.

Plotting the histogram for the APC values of AD and CN groups separately for all 360 ROIs (irrespective of statistical significance) revealed a clear group separation. Histograms of AD groups were clearly shifted to left compared to CN group for cortical volume, cortical area and cortical thickness as measured by longitudinal SBM (Figures 6A–C). By contrast, the histograms of AD and CN group for cortical gyrification and depth APC values did much more overlap and were not clearly separable (Figures 6D,E).

### Longitudinal Surface Based Morphometry Optimally Suitable for Storey-Tibshirani Multiple Testing Correction

We applied the Storey-Tibshirani method (Storey and Tibshirani, 2003) for multiple testing correction because the distribution of individual *p*-values perfectly meets the requirements with a large set of individual *p*-values, a peak of the *p*-value distribution at low values and a flat plateau for high *p*-values close to 1 (Supplementary Figure 2). Consequently, this method delivered a more appropriate approximation of FDR than Benjamini–Hochberg approach and more ROIs remained significant after multiple testing correction (Table 3).

### Cognitive Decline

**FIGURE 7:**
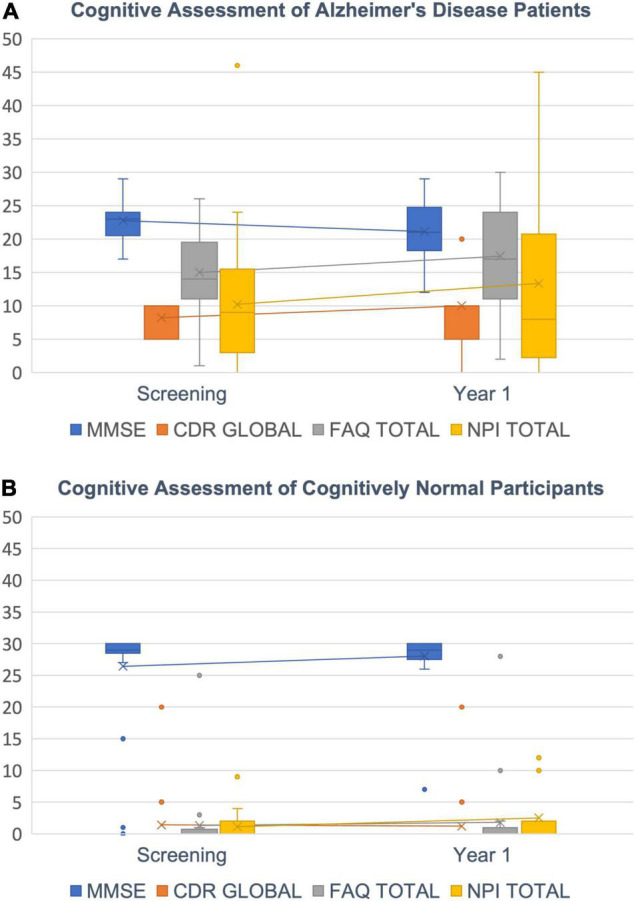
Cognitive decline. **(A)** AD Patients’ decrease in cognitive abilities is indicated by a decreasing MMSE score (blue) and increasing CDR global (orange), FAQ total (gray) and NPI total scores (yellow). **(B)** CN subjects show scores around 30 for MMSE and close to zero for CDR global, FAQ total and NPI total as expected. Box plots with median (horizontal lines) percentiles (boxes), standard deviations (error bars) and outliers (dots).

A worsening of cognitive performance was seen in the majority of AD patients over a time course of 1 year but not in age matched CN subjects. We saw a pronounced decrease in Mini-Mental-State Exam (MMSE; Folstein et al., 1975) in most AD patients but only a small or no decrease in most CN subjects. For Clinical Dementia Rating global score (CDR global; Berg et al., 1988), Functional Activities Questionnaire total score (FAQ total; Pfeffer et al., 1982) and Neuropsychiatric Inventory total score (NPI total; Cummings et al., 1994) we found an increase in the respective scores in patients (Figure 7). The variance of those test results is typically quite high across patients and was greatest for NPI total scores.

We further correlated the change in cognitive scores with measured changes in cortical morphology. For this, we calculated Spearman rank correlations between changes in the four cognitive scores and APC values for volume (VBM, SBM), cortical thickness and area (SBM) for all ROIs. Although correlations are not very strong, we find a clear pattern for ROIs with a significant decay in volume (Table 4): APCs of VBM-derived volume and SBM-derived feature changes (Volume, Area, Thickness) were positively correlated to MMSE and negatively to CDR global, FAQ total and NPI total. In other words, the loss in brain morphology correlates with a worsening of cognitive scores in AD patients. Importantly, this effect does not extend to the entirety of ROIs with non-significant APCs. In other words, if APCs are significant, they correlate in the correct direction in almost all cases. If they are not significant there is a mix of positive and negative correlations.

**TABLE 4:** Correlation between morphometric and cognitive changes.

ROI	Measure	Q	cor w/MMSE	cor w/CDR global	cor w/FAQ total	cor w/NPI total	
L_Hippocampus	Volume	0.0004	0.16	−0.34	−0.27	−0.25	VBM
R_Hippocampus	Volume	0.0054	0.10	−0.29	−0.27	−0.03	
L_Amygdala	Volume	0.0182	0.39	−0.22	−0.46	−0.18	
L_VVC	Volume	0.0028	0.07	−0.30	−0.25	−0.04	SBM
L_PH	Volume	0.0065	0.11	−0.16	−0.31	−0.27	
R_TGd	Volume	0.0165	0.32	−0.45	−0.24	−0.10	
R_PFm	Volume	0.0165	0.49	−0.36	−0.20	0.04	
L_TGd	Volume	0.0165	0.44	−0.36	−0.12	−0.10	
L_PF	Volume	0.0172	0.25	−0.40	−0.36	−0.31	
L_TF	Volume	0.0203	0.09	−0.44	−0.44	−0.17	
L_TE1p	Volume	0.0206	0.17	−0.27	−0.26	−0.13	
L_A4	Volume	0.0287	0.28	−0.52	−0.26	−0.20	
L_FST	Volume	0.0287	0.13	−0.19	−0.46	−0.21	
L_TE1a	Volume	0.0287	0.39	−0.22	−0.43	−0.20	
L_PeEc	Volume	0.0311	0.21	−0.42	−0.38	−0.09	
R_TE2a	Volume	0.0325	0.40	−0.42	−0.27	0.16	
R_EC	Volume	0.0401	0.24	−0.18	−0.09	−0.09	
L_FFC	Volume	0.0401	0.03	−0.31	−0.37	−0.08	
R_EC	Area	0.0165	0.29	−0.37	−0.25	−0.12	
L_9a	Area	0.0206	0.25	−0.16	−0.09	−0.07	
L_PH	Area	0.0357	0.05	−0.04	−0.02	0.07	
R_33pr	Area	0.0401	0.11	−0.22	−0.16	−0.10	
L_IFJa	Area	0.0401	0.07	−0.12	−0.17	−0.13	
R_s6-8	Thickness	0.0165	0.17	−0.25	−0.16	−0.16	
L_TE2p	Thickness	0.0325	−0.01	−0.19	−0.32	−0.08	

*Each line reports correlations between one ROI from either VBM or SBM and cognitive scores MMSE, CDR global, FAQ total, NPI total. Q values indicate significance levels for morphometry group comparisons and are copied from Tables 2, 3.*

## Discussion

In this paper we reported stronger longitudinal brain morphometry loss corresponding to an accelerated cognitive decline in AD patients over 1 year of time compared to matched CN subjects. AD patients showed a stronger decrease in cortical volume, area and thickness over the course of 1 year measured by SBM. Longitudinal SBM assessments complemented longitudinal VBM measurements and increased the number of significant ROI comparisons including temporal areas, anterior cingulate areas and frontal regions—whereas longitudinal VBM only detected significant longitudinal changes in hippocampus and amygdala. Moreover, the distribution of local regional changes as reported by SBM allowed for a clear separation between the two groups. Our results are in line with previous studies demonstrating that SBM can strengthen longitudinal assessments in aging and neurodegeneration studies (Tustison et al., 2019).

### Toward a Gold-Standard for Longitudinal MRI-Based Morphometry Assessments

Our current approach is the first longitudinal morphometry assessment that combines high resolution imaging (from ADNI3 study; Weiner et al., 2017) and a high-resolved brain atlas (HCP_MMP_1.0; Glasser et al., 2016) with a mathematical sound multiplicative annual percent change model and an adequate multiple testing correction adopted from genome-wide association studies (Storey and Tibshirani, 2003). Combining all these methodological aspects was required to arrive at a statistical significance level that would be required for clinical trials. Previous approaches often fall short in actually detecting significant longitudinal morphometry differences because any of the afore-mentioned steps left uncontrolled may cause inaccuracies preventing significance.

### High-Resolution Image Assessments Require High Image Quality

We have only chosen subjects from ADNI-3 study because it is the largest multi-site, multi-vendor study to leverage several advanced MRI methods (Weiner et al., 2017). Providing high-resolution medial temporal lobe (MTL) subregion imaging offers quantification of changes in hippocampal subfields and parahippocampal gyrus subregions, which are the location of the earliest stages of tau pathology (Pluta et al., 2012; Mueller et al., 2013; de Flores et al., 2015; Yushkevich et al., 2015). All ADNI-3 scans were acquired at 3T. High image quality is essential for performing high-resolution image assessments. Especially for surface reconstructions needed for SBM, high image quality is needed to exactly determine gray and white matter borders. Moreover, with the HCP MMP 1.0 brain atlas (Glasser et al., 2016) we applied one of the latest multimodal brain atlases which generates a robust neuroanatomical map of human neocortical areas in any individual. Highly reproducible and generalizable cortical parcellation of the cortex becomes possible through state-of-the-art methods of data acquisition, preprocessing, and analysis designed to compensate for individual variability and thereby minimize blurring of images. The situation is analogous to astronomy in which ground-based telescopes produced relatively blurry images of the sky before the advent of adaptive optics and space telescopes (Glasser et al., 2016).

### Annual Percent Change as a Measure for Longitudinal Effects

In spite of high image acquisition and assessment quality, varying observation intervals in longitudinal studies can though be a considerable source of unavoidable inconsistencies and increasing variances: Although the study design aims at re-assessing subjects after, e.g., 12 months, in reality study participants return for a second visit not exactly after 12 months and differences in longitudinal observation intervals can often differ quite substantially and need to be corrected for statistical evaluation. Considering the APC has proven as a suitable way (Jockwitz et al., 2021, Front Human Neurosci) to correct differences in longitudinal observation intervals within a certain range. By using the APC, we assumed a quasi-continuous brain change in the time difference between the planned and the observed interval. Obviously, this assumption is not necessarily fulfilled if this difference becomes too large for the individual subject and the subject might need to be excluded from the longitudinal assessment. If the study uses a different follow-up design, e.g., of 180 days instead of 365, the formula needs to be adapted accordingly in order to normalize to 180 days. We may draw the reader’s attention to the fact that continuous percentage changes as assumed for the APC behave multiplicatorily. This mathematical property leads to the correct APC formula above while additive equations for APC would be a mathematically incorrect over-simplification leading to an under or over-correction of the influence of the different observed longitudinal intervals.

### The Need for an Appropriate Multiple Testing Correction

Multiple tests especially pose a challenge in brain morphometry assessments because studies typically have a comparably low sample size, often only moderate effect sizes but at the same time a high number of statistical tests due to a large number of ROIs. This situation very often leaves no statistical significances after multiple testing correction. Paradoxically, the more fine-grained the assessment becomes by higher number of ROIs, the more pessimistic multiple testing correction will be. Fortunately, the high number of comparisons can be used for a very precise fitting of the *p*-value distribution and the Storey-Tibshirani approach for multiple testing correction (Storey and Tibshirani, 2003) becomes most powerful resulting in more significant comparisons. Moreover, by taking the distribution of *p*-values into account, the false discovery rate becomes controllable. In our case, we may conclude that with a false discovery rate of 5% only one out of 22 ROI features could be erroneously assumed to be statistically different although it is in fact not.

### Temporal Cortex Shows Strongest Morphometry Decay in Alzheimer’s Disease Patients

Even though there is a ubiquitous and quite consistent trend of brain morphometry loss in wide parts of the cortex, the temporal lobe expressed the most significant effects even after multiple testing correction. Our findings are well in line with previous literature showing that the medial temporal lobe degenerates strongest in AD patients compared to typically aging subjects (Jack et al., 1998; Chauveau et al., 2021). Here, we documented temporal tissue loss by more precise surface based morphometry and at a higher resolution of 360 cortical areas, which has become possible due to increased imaging quality in ADNI3 compared to previous ADNI studies (Jack et al., 2008) and further developed software-aided image assessments. In line with a previous study, we found temporal areas to be differently affected by cortical thickness loss and cortical surface area loss (Iannopollo and Garcia, 2021). High precision evaluation of temporal lobe atrophy is of particular importance as temporal cortex thinning is associated with episodic memory impairments (Das et al., 2016) and even depression (Fujishima et al., 2014) while temporal volume loss was found in healthy aging subjects (Fjell et al., 2009) and may be a potential early indicator of an increased risk for developing AD later in life. At least, an increased temporal volume loss in MCI patients was discussed as a predictor for the conversion from MCI to AD (Jack et al., 1999).

### Visual Cortex Mostly Spared From Neurodegeneration in Alzheimer’s Disease Patients

We saw that the visual cortex was mostly spared from neurodegeneration as almost no ROIs of the visual cortex in AD group showed significant differences in longitudinal changes compared to CN controls. Some statistically significant effects were seen in higher multimodal areas of the occipital lobe for example in the parieto-occipital sulcus or at the temporo-parietal-occipital junction (TPOJ). The brain region at the TPOJ has been discussed in the context of highest cognitive functions including integration of external and body information (Abu-Akel and Shamay-Tsoory, 2011), making moral decisions (Blanke and Arzy, 2005) and theory of mind (Saxe and Kanwisher, 2003) and is well known to be affected by AD (Coughlan et al., 2018). By contrast, the visual cortex is affected relatively late in the course of the disease (Albers et al., 2015 but see also Wu et al., 2020). Therefore, our longitudinal results are in line with current knowledge about brain atrophy in AD.

### Limitations of Imaging Results

Even with highest image processing routines uncertainties remain. For example, we see a stronger decay in hippocampal tail volume in CN subjects compared to AD patients that cannot be fully explained. However, from post-mortem studies a greater variability in hippocampal tail volume is well known (Adler et al., 2018). Caused by the bending of the tail, a greater variability is observed in the appearance of the hippocampal tail in the coronal plane. When sectioning the tail in the direction of the tail bend, the tail has a body-like structure in all subjects. This information could potentially explain variations of *in vivo* segmentations of the tail, which is generally omitted from segmentation protocols due to perceived anatomical complexity (Adler et al., 2018).

Brain morphometry decay and scores indicating cognitive decline are correlated as expected. However, due to limited sensitivity and test re-test reliability of cognitive scores, correlations cannot be expected to be higher. At least, the signs of the correlations are as expected for significantly stronger decreasing ROIs in AD patients but are not consistent for non-significant ROIs. This overall finding strengthens the outcome of the present work that accelerated regional decay can be attributed to AD relevant clinical observations but larger group sizes would be required to obtain stronger correlations.

In face of optimal image processing and statistical assessment, the relatively low numbers of AD vs. matched CN participants that were included in this study, still remain a challenge. ADNI participants discontinuing follow-up assessments and inconsistent image quality, mostly brought about by head movements, could not fully be corrected in all originally available subjects eventually leading to such a small group size. A consistent high MR image quality in a clinical setting would improve neuroimaging research quality at the initial stage, and ultimately better the therapeutical outcome to the individual patient.

## Conclusion

We could show that even with low study size, reliable longitudinal group effects could be obtained for a high number of cortical ROIs when high MR imaging quality, most advanced image segmentation and surface based morphometry is used in combination with correct APC values and appropriate statistical testing. Our compelling approach not only paves the way for developing earlier biomarkers for AD, the high number of ROIs and also the enhanced separability between the two groups by SBM may also be suitable to detect longitudinal changes in response to pharmacological treatment more accurately. The complete workflow from image processing for feature extraction to statistical assessments can be fully automated in NICARA, which makes the approach particularly attractive for larger cohorts.

## Data Availability Statement

Publicly available datasets were analyzed in this study. This data can be found here: Alzheimer’s Disease Neuroimaging Initiative.

## Ethics Statement

The studies involving human participants were reviewed and approved by Alzheimer’s Disease Neuroimaging Initiative. The patients/participants provided their written informed consent to participate in this study.

## Author Contributions

SR developed the statistical model, did all statistical tests, and wrote the manuscript. YL developed the image processing streams, did the image analysis and the manual image quality control, and wrote the manuscript. SK did the NICARA implementations. MB-O designed the study and wrote the manuscript. All authors contributed to the article and approved the submitted version.

## Conflict of Interest

SR was employed by company Viscovery Software GmbH. SR, YL, SK, and MB-O were employed with Biomax or subsidiaries of Biomax. Viscovery Software GmbH is a one hundred percent subsidiary of Biomax Informatics.

## Publisher’s Note

All claims expressed in this article are solely those of the authors and do not necessarily represent those of their affiliated organizations, or those of the publisher, the editors and the reviewers. Any product that may be evaluated in this article, or claim that may be made by its manufacturer, is not guaranteed or endorsed by the publisher.

## Footnotes

1
http://adni.loni.usc.edu/wp-content/uploads/2008/07/adni2-procedures-manual.pdf

2
https://nicara.eu

3
http://www.neuro.uni-jena.de/cat/

4
http://www.fil.ion.ucl.ac.uk/spm/software/spm12/

5
https://surfer.nmr.mgh.harvard.edu/fswiki/FreeSurferWiki

## Supplementary Material

Supplementary Figure 1 Histogram of *p*-values from individual *t*-test for longitudinal VBM. The distribution of *p*-values from longitudinal VBM peaked for small *p*-values but did not show a plateau for larger *p*-values. With this, not all requirements for Storey-Tibshirani multiple testing correction method (Storey and Tibshirani, 2003) were fulfilled. Hence, Storey-Tibshirani correction was comparably pessimistic as Benjamini and Hochberg (1995) multiple testing correction.

Supplementary Figure 2 Histogram of *p*-values from individual *t*-test for longitudinal SBM. The distribution of *p*-values from longitudinal VBM peaked for small *p*-values and showed a plateau for larger *p*-values. Moreover, the 360 individual *p*-values from individual tests allowed for a small bin size for an accurate resembling of the distribution. With this, all preconditions for Storey-Tibshirani approach (2003) are fulfilled and more ROI comparisons remain significant after multiple testing correction.

Supplementary Table 1 Demographic tabular listing with APOE genotypes. For each subject, the table lists two rows, one per session (Screening and Year 1), with columns Age Range, Age, Sex, Diagnosis, MMSE scores, APOE A1 and A2, CDR GLOBAL score, FAQ TOTAL score, NPI TOTAL. Values for ABETA40, ABETA42, PTAU and TAU are available for some subjects but overall largely incomplete and are therefore excluded from any statistical assessment.

Supplementary Table 2 360 Regions of Interests as defined by the HCP MMP 1.0 atlas. Each row contains the abbreviation and full name for each ROI as well as hemisphere and a part of information derived from neurosurgical literature (Baker et al., 2018).
