# Supplementary figures and images for: Automated High-Definition MRI Processing Routine Robustly Detects Longitudinal Morphometry Changes in Alzheimer’s Disease Patients
Source: Front Aging Neurosci. 2022 Jun 7;14:832828. doi: 10.3389/fnagi.2022.832828 (PMC9211026; doi:10.3389/fnagi.2022.832828)

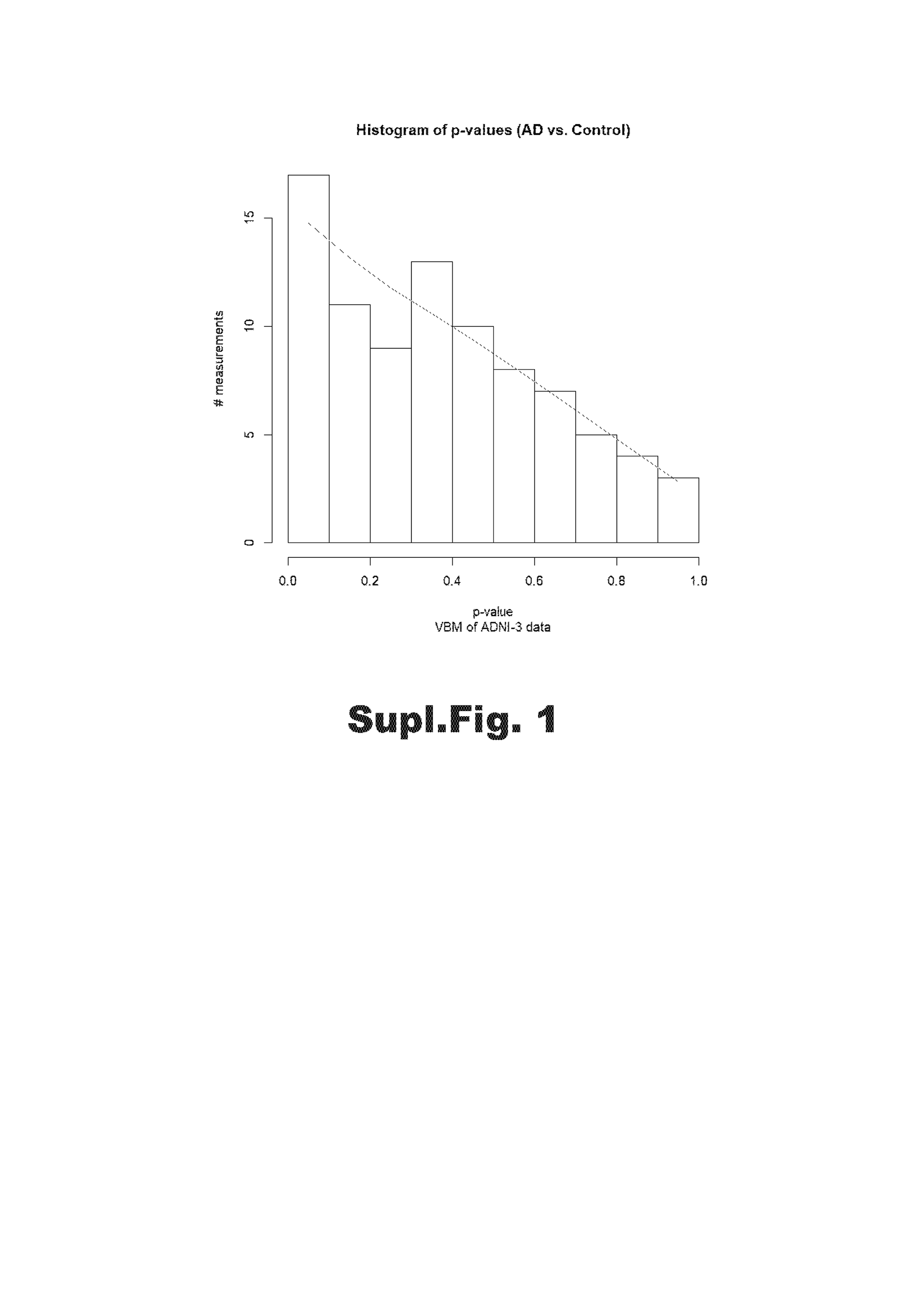

Supplement: Supplementary Figure 1 — Histogram of p-values from individual t-test for longitudinal VBM. The distribution of p-values from longitudinal VBM peaked for small p-values but did not show a plateau for larger p-values. With this, not all requirements for Storey-Tibshirani multiple testing correction method (Storey and Tibshirani, 2003) were fulfilled. Hence, Storey-Tibshirani correction was comparably pessimistic as Benjamini and Hochberg (1995) multiple testing correction. [file Image_1.TIF]

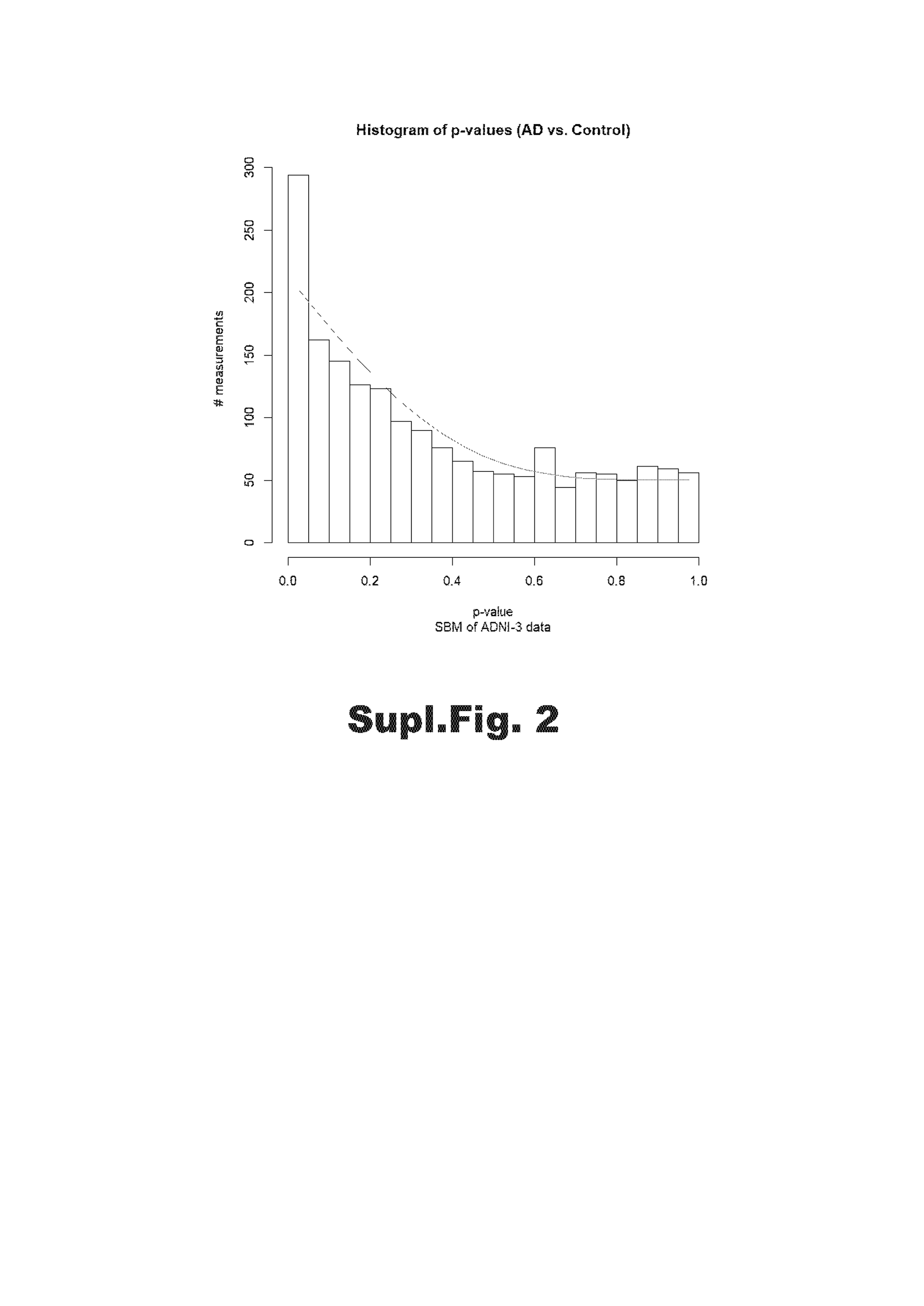

Supplement: Supplementary Figure 2 — Histogram of p-values from individual t-test for longitudinal SBM. The distribution of p-values from longitudinal VBM peaked for small p-values and showed a plateau for larger p-values. Moreover, the 360 individual p-values from individual tests allowed for a small bin size for an accurate resembling of the distribution. With this, all preconditions for Storey-Tibshirani approach (2003) are fulfilled and more ROI comparisons remain significant after multiple testing correction. [file Image_2.TIF]
